# Thirty-year transition of disease burden attributed to low bone mineral density: A disaggregated analysis of Global Patterns and China’s Epidemiological Profile (1990–2021)

**DOI:** 10.1097/MD.0000000000049316

**Published:** 2026-06-12

**Authors:** Leiming Ge, Zhijuan Qi, Yao Liu, Ling Shi, Jianling Song, Shuping Zheng, Xiaodong Wang, Xiaopan Li, Wenchang Jia, Zheng Ye

**Affiliations:** a Department of General Practice, Changfeng Community Health Service Center, Shanghai, China; b Department of General Practice, Shiquan Community Health Service Center, Shanghai, China; c Department of General Practice, Zhongshan Hospital, Shanghai Medical College of Fudan University, Shanghai, China; d Shanghai Putuo District Health Affairs Management Centre, Shanghai, China; e Department of Health Management Center, Zhongshan Hospital, Shanghai Medical College of Fudan University, Shanghai, China; f School of Public Health, Fudan University, Shanghai, China.

**Keywords:** disability-adjusted life years, global burden, low bone mineral density, socio-demographic indices

## Abstract

This study aims to compare the disease burden of low bone mineral density (LBMD) between China and global populations from 1990 to 2021 and quantify the associations of sex, age, and socio-demographic index with disability-adjusted life years (DALYs). A secondary analysis of LBMD was conducted using comprehensive data from the Global Burden of Disease Study 2021. The absolute number of DALYs and DALYs rates were determined for 1990 and 2021. Joinpoint regression analysis was employed to assess trends in the age-standardized DALYs rate from 1990 to 2021 and over the most recent decade (2012–2021), using the average annual percentage change. Additionally, China’s future trends over the next 20 years were projected based on this analysis. In 2021, LBMD accounted for 17.31 million global DALYs and 3.47 million (95% UI: 2.78–4.24) in China. Despite a decline in China’s age-standardized rate (from 195 to 175 per 1,00,000; average annual percentage change = −0.29%; 95% CI: −0.32 to −0.25), the absolute DALYs more than doubled. The most pronounced increase occurred in adults aged ≥80 years, with older women exhibiting rates 22.5% higher than men. Projections indicate a rise in China’s rate from 175 to 210 per 1,00,000 by 2050, starting in approximately 2040. Women, older adults, and populations in low-middle and middle socio-demographic index regions are high-risk groups that require prioritized attention. These findings highlight the need for age-stratified screening protocols and context-specific resource allocation in low-middle SDI regions.

## 1. Introduction

Bone mineral density (BMD), quantified through standardized dual-energy X-ray absorptiometry (sBMD) at the femoral neck (g/cm^2^), is the principal biomarker for assessing skeletal integrity.^[1]^ Its Gaussian distribution in healthy populations enables diagnostic categorization via standard deviation units relative to young adult reference values, with the World Health Organization defining osteopenia as BMD 1.0 to 2.5 standard deviation below this threshold (T-score: −1 to −2.5).^[1]^ The Global Burden of Disease (GBD) 2021 framework further operationalizes low bone mineral density (LBMD) through age- and sex-specific 99th percentile comparisons (theoretical minimum-risk exposure level), establishing population-attributable burden estimates for adults aged ≥20 years.^[1]^ Clinically asymptomatic until fracture manifestation, LBMD significantly elevates the risk of osteoporotic fractures, falls, and malignancy in aging cohorts.^[2,3]^ These events generate substantial socioeconomic burdens, including direct healthcare costs estimated at billions of dollars annually, as well as indirect costs from prolonged work absenteeism, reduced productivity, and increased disability claims.^[4-6]^ In particular, these challenges are magnified in rapidly aging societies like China, where 14.2% of the population exceeded 65 years of age by 2021 – a demographic shift demanding urgent epidemiological scrutiny.^[7]^

LBMD pathogenesis arises from a multifactorial etiology, including age,^[8]^ hormonal shifts,^[9]^ modifiable determinants such as dietary patterns,^[10]^ and environmental exposures.^[11]^ While prevalence escalates markedly after age 40, critical knowledge gaps persist regarding male-specific epidemiology^[12]^ and socio-demographic index (SDI)-stratified trends.^[9]^ Although the Global Burden of Disease Study 2021 (GBD 2021) provides essential frameworks for cross-population burden estimation,^[2,13]^ contemporary data remain scarce post-2019, with the potential impact of the COVID-19 pandemic on LBMD trajectories yet to be quantified. A granular analysis encompassing age, sex, and SDI stratification is imperative to optimize targeted intervention strategies in diverse populations.

This study aimed to compare temporal trends in age-standardized disability-adjusted life years (DALYs) attributable to LBMD across SDI quintiles from 1990 to 2021, and to develop Bayesian hierarchical models for projecting China’s LBMD-related disease burden through 2050. These findings are expected to provide crucial insights that can inform policy development and clinical practice in the future.

## 2. Materials and methods

### 2.1. Data source and access

This study utilized the publicly available GBD 2021 database, which was administered by the Institute for Health Metrics and Evaluation, to assess disease burden, harm, and risk factors across 204 countries and territories, including specific subnational locations. It encompasses data on years lived with disability (YLD), DALYs, and Healthy Life Expectancy from 1990 to 2021.^[13]^ This resource is instrumental in identifying health disparities among diverse populations worldwide, tracking temporal changes, evaluating health progress, and developing strategies to address health inequities in the post-COVID-19 era.^[14]^

### 2.2. Variables

#### 2.2.1. The burden of LBMD

DALYs were used to quantify the burden of LBMD. DALYs measure the loss of healthy life in years and consist of 2 components^[15,16]^: years of life lost, reflecting premature mortality,^[17]^ and YLD, representing the time spent in suboptimal health due to a condition.^[18]^ The sum of YLL and YLD provides the total number of DALYs, offering a comprehensive assessment of the disease burden by integrating mortality and morbidity data. This metric is a reliable tool for evaluating the impact on population health and informing policy and clinical decisions.

The DALYs rate, expressed per 1,00,000 individuals, standardizes the disease burden across populations. A higher DALYs rate indicates a greater health impact of a disease, enabling comparisons across regions, countries, and demographic groups. Analyzing trends in the DALYs rates over time helps assess changes in the disease burden, supporting public health planning and intervention strategies.

The age-standardized DALYs rate further refines this measure by adjusting for differences in the population age structure, allowing for unbiased comparisons of disease burden across regions, countries, and time periods. By applying the standard age distribution, the age-standardized DALYs rate eliminates demographic variations, making it a robust tool for evaluating health impacts in diverse populations.

The average annual percentage change (AAPC) quantifies the annual rate of change in a variable over a specified period. In this study, the AAPC represents the yearly percentage change (increase, decrease, or stability) derived from the weighted average slope of the joinpoint regression model from 1990 to 2021.^[19]^

### 2.2.2. Socio‑demographic index

The SDI is a comprehensive measure of a country’s or region’s social and economic factors that influence health outcomes. This index is computed as a geometric mean ranging from 0 to 1, incorporating 3 key elements: per capita income, average educational attainment of individuals aged 15 and above, and fertility rates among women aged under 25 years.^[20]^ The SDI is categorized into 5 distinct levels: high SDI (exceeding 0.81), high-middle SDI (0.70–0.81), middle SDI (0.61–0.69), low-middle SDI (0.46–0.60), and low SDI (below 0.46).^[15]^

### 2.2.3. Statistical analysis

Annual cases and age-standardized rates with uncertainty intervals (UI) for DALYs of LBMD from 1990 to 2021 were grouped by sex, age, and location.

Upon applying joinpoint regression analysis to log-transform data for all trend studies, the Grid Search technique, Monte Carlo permutation tests, and Modified Bayesian Information Criterion were used to display the optimum yearly trend curve and its variance. An aggregate measure of trend over a predetermined fixed interval – in this study, the whole year (1990–2021) – was the average yearly percentage change. When the lower limit of the 95% confidence interval (CI) was greater than 0, the trend of significance increased; when the upper limit of the CIs was less than 0, the trend of significance decreased.

Employing integrated nested Laplace approximations (INLA) and the R-package of Bayesian age-period-cohort (BAPC) and INLA, the BAPC model was used to predict future trends for the prevalence and DALYs of LBMD from 2021 to 2050 based on the available data. All data were analyzed using R software (version 4.1.2; MathSoft, Cambridge).

## 3. Results

### 3.1. Absolute number of DALYs for LBMD in global, China, and SDI regions

From 1990 to 2021 (Table 1), the global burden of LBMD, measured in absolute terms of DALYs, increased from 90,87,697.74 to 1,73,10,084.57 (95% UI: 1,42,94,591 to 2,05,11,120). During the same period, China saw a similar rise, with LBMD-related DALYs growing from 15,06,204.77 to 34,65,540.42 (95% UI: 27,76,245 to 42,37,543). Notably, these values represent absolute DALYs and do not account for changes in population size or structure over time.

**Table 1:** Absolute number of DALY for LBMDs in China, global, by SDI region.

China/global/regions	Both		Male		Female	
	Number	95% UI	Number	95% UI	Number	95% UI
China						
1990	15,06,204.77	(12,41,034.38, 17,86,388.93)	7,97,528.08	(6,36,481.18, 9,50,378.08)	7,08,676.69	(5,72,722.95, 8,65,635.81)
2019	32,90,623.00	(26,39,287.98, 40,37,712.61)	17,00,256.39	(13,24,239.09, 21,34,290.70)	15,90,366.61	(12,63,788.98, 20,22,423.00)
2020	34,26,122.49	(26,65,953.32, 42,04,734.37)	17,47,619.86	(13,35,426.76, 21,59,897.48)	16,78,502.64	(12,82,095.50, 20,88,548.87)
2021	34,65,540.42	(27,76,245.48, 42,37,543.94)	17,52,598.48	(13,85,994.22, 21,73,150.67)	17,12,941.93	(13,08,981.72, 21,39,614.43)
Global						
1990	90,87,697.74	(75,23,812.22, 1,06,21,387.95)	44,88,970.38	(37,50,974.21, 51,89,584.76)	45,98,727.36	(37,70,743.00, 55,22,284.00)
2019	1,66,98,263.46	(1,37,42,441.46, 1,98,81,123.80)	79,71,892.33	(67,03,690.46, 92,55,920.68)	87,26,371.12	(70,84,709.23, 1,06,51,010.64)
2020	1,70,61,630.60	(1,41,32,337.80, 2,03,37,259.03)	81,26,669.94	(67,51,456.04, 93,88,206.91)	89,34,960.66	(73,03,955.34, 1,09,52,769.51)
2021	1,73,10,084.57	(1,42,94,591.61, 2,05,11,120.41)	82,18,289.78	(68,94,568.34, 95,89,722.23)	90,91,794.79	(73,46,620.46, 1,11,68,005.08)
High SDI						
1990	25,23,190.12	(19,97,977.58, 30,97,979.80)	10,49,579.00	(8,44,430.69, 12,69,241.85)	14,73,611.12	(11,43,536.66, 18,51,302.37)
2019	41,05,144.73	(32,45,672.42, 51,30,114.67)	16,82,203.61	(13,76,435.33, 20,27,398.45)	24,22,941.12	(18,74,321.39, 31,03,444.79)
2020	41,66,033.07	(32,86,052.11, 52,17,065.80)	17,06,045.46	(13,96,647.01, 20,63,109.68)	24,59,987.61	(18,91,258.89, 31,67,090.15)
2021	42,21,345.78	(33,15,858.94, 52,66,246.95)	17,30,235.95	(14,10,935.87, 20,86,588.18)	24,91,109.84	(19,11,105.47, 31,94,362.70)
High-middle SDI						
1990	22,04,336.48	(17,72,743.24, 26,41,165.69)	11,02,743.95	(8,95,886.38, 13,13,572.98)	11,01,592.53	(8,80,208.05, 13,64,163.89)
2019	32,88,150.17	(26,36,391.74, 40,80,746.98)	15,91,868.12	(12,79,956.24, 19,36,750.77)	16,96,282.05	(13,31,260.59, 21,40,985.05)
2020	33,68,391.03	(27,07,513.97, 41,50,367.66)	16,21,035.67	(13,00,876.78, 19,39,314.17)	17,47,355.37	(13,68,342.55, 22,24,570.30)
2021	33,97,696.79	(27,36,433.30, 42,19,222.24)	16,26,270.34	(1,32,39,61.14, 19,82,170.15)	17,71,426.44	(13,84,074.98, 22,66,226.62)
Middle SDI						
1990	22,61,679.04	(19,27,758.34, 25,64,283.63)	12,36,655.37	(10,42,218.25, 14,08,955.16)	10,25,023.67	(8,64,574.59, 12,01,608.49)
2019	47,90,460.62	(40,27,605.36, 5,64,1228.34)	24,75,164.85	(20,83,426.02, 28,85,017.42)	23,15,295.77	(19,22,805.44, 27,78,864.44)
2020	49,35,368.34	(40,95,155.26, 58,06,972.11)	25,34,339.34	(20,81,254.53, 29,16,307.42)	24,01,029.01	(19,41,915.29, 28,88,583.40)
2021	50,33,128.69	(42,24,093.24, 59,44,699.67)	25,68,591.09	(21,76,102.93, 29,78,208.19)	24,64,537.59	(20,145,18.50, 29,72,171.01)
Low-middle SDI						
1990	14,93,619.29	(12,77,979.42, 16,89,633.47)	7,61,681.98	(6,56,997.21, 8,58,148.29)	7,31,937.31	(6,03,447.57, 8,60,948.91)
2019	33,12,728.92	(28,43,065.17, 37,45,686.01)	15,89,787.01	(13,64,925.94, 17,82,512.12)	17,22,941.91	(14,51,351.70, 20,17,334.25)
2020	33,64,829.19	(29,03,008.72, 38,17,355.70)	16,17,530.73	(13,81,171.36, 18,10,275.51)	17,47,298.46	(14,66,927.29, 20,46,384.27)
2021	34,08,075.87	(28,86,442.59, 38,76,362.25)	16,36,812.77	(13,95,667.53, 18,52,879.63)	17,71,263.09	(14,69,689.49, 20,61,802.37)
Low SDI						
1990	5,93,093.35	(5,15,034.82, 6,64,374.72)	3,32,638.88	(2,85,622.32, 3,78,483.31)	2,60,454.48	(2,16,456.59, 3,02,068.96)
2019	11,87,397.52	(10,28,194.16, 13,39,168.84)	6,25,836.03	(5,38,281.20, 7,04,437.96)	5,61,561.49	(4,66,901.66, 6,44,714.87)
2020	12,12,422.70	(10,44,255.96, 13,69,973.27)	6,40,587.75	(5,51,455.00, 7,28,214.79)	5,71,834.95	(4,77,304.37, 6,61,623.99)
2021	12,35,176.06	(10,59,938.57, 13,87,726.60)	6,49,238.30	(5,47,178.61, 7,32,743.17)	5,85,937.76	(4,90,127.23, 6,82,600.99)

DALY = disability-adjusted life year, LBMD = low bone mineral density, SDI = socio-demographic index, UI = uncertainty interval.

Globally, women bore a higher burden of absolute DALYs in 2021, with females accounting for 90,91,794.79 (95% UI: 73,46,620–1,11,68,005) compared with 82,18,289.78 (95% UI: 68,94,568–95,89,722) for males. In contrast, China displayed an inverse pattern, with men exhibiting higher absolute DALYs (17,52,598.48; 95% UI: 13,85,994.22 to 21,73,150.67) than women (17,12,941.93; 95% UI: 13,08,981.72 to 21,39,614.43) in 2021. A similar male-predominant trend was observed in the Middle SDI region (difference: +1,04,053.50) and the low SDI region (difference: +63,300.54). Conversely, the low-middle SDI region demonstrated a female-predominant pattern, with women carrying a higher burden (difference: −1,34,450.32).

In 2021, the middle SDI region recorded the highest DALYs at 50,33,128.69, followed closely by the high SDI region with 42,21,345.78 DALYs. In contrast, low-middle SDI areas, though beginning with a lower baseline near 1.5 million DALYs in 1990, experienced one of the most marked increases over the period, reaching approximately 3.4 million DALYs by 2021. Similarly, the high-middle SDI group showed substantial growth, increasing from 2.2 million to nearly 3.4 million DALYs. Even low-SDI regions, despite having the smallest absolute burden, experienced a gradual rise, with the number of DALYs increasing from approximately 0.59 million in 1990 to over 1.2 million in 2021, reflecting a persistent upward trajectory across all SDI groups.

### 3.2. Gender and age-specific trends in DALYs rate (1990 vs 2021)

**Figure 1.**
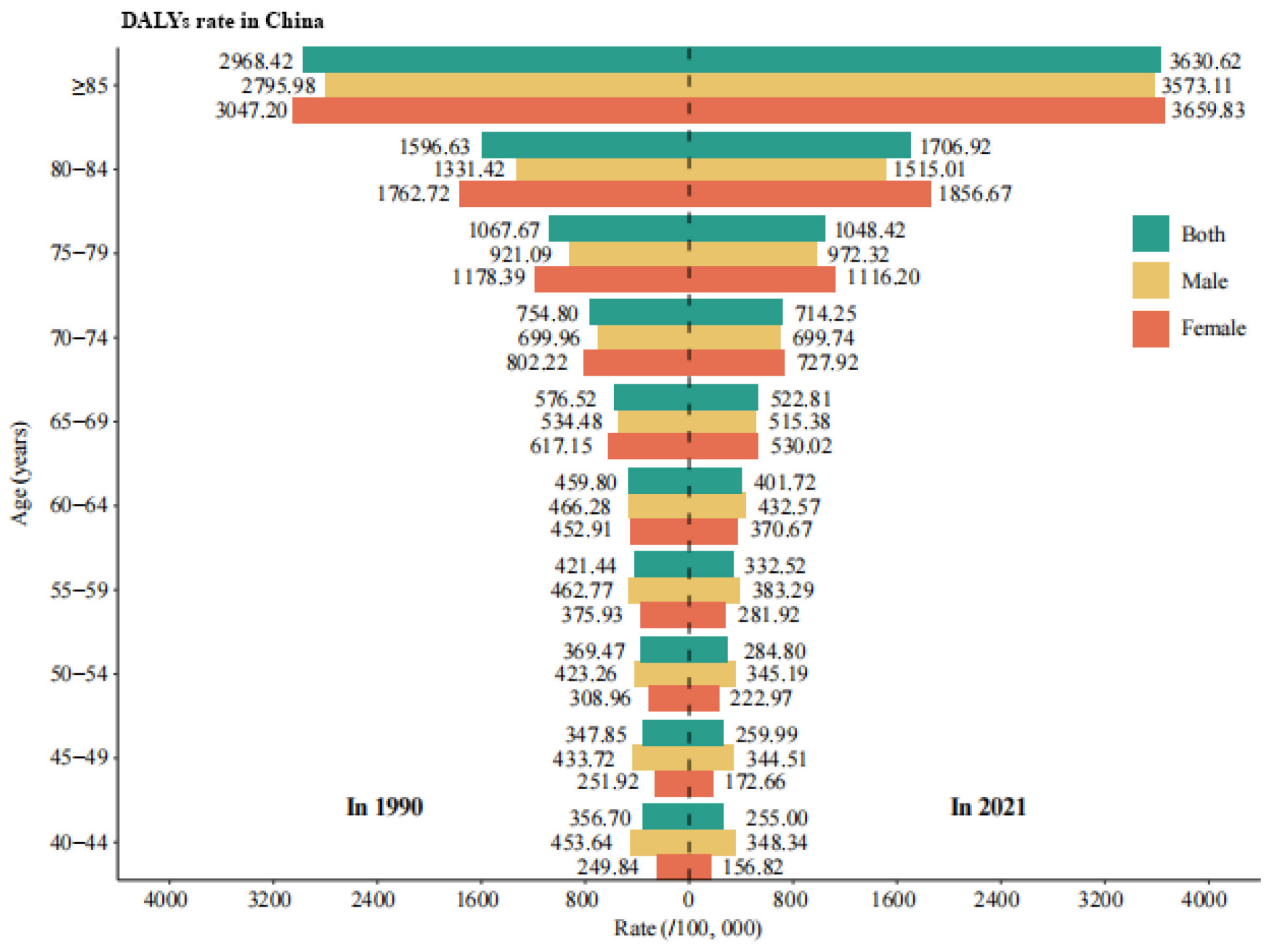
Presents the sex- and age-specific disability-adjusted life years (DALYs) rates for diseases related to low bone mineral density (LBMD) in China, comparing data from 1990 and 2021. The stacked bars represent the total DALYs (green), with contributions from males (yellow) and females (orange) clearly distinguished, allowing for a direct comparison of the burden distribution between the 2 time points and demographic groups. DALY = disability-adjusted life year, LBMD = low bone mineral density.

According to 2021 statistics (Fig. 1), the DALYs rate exhibited a clear age-dependent pattern, with rates progressively increasing with age. Notably, the most pronounced increase was observed in individuals aged ≥85 years. When categorizing the population into 2 distinct groups – middle-aged individuals (<65 years) and older adults (≥65 years) – clear sex-based disparities in DALYs were identified. Within the middle-aged group, males demonstrated a higher DALYs rate than females. Conversely, in the older adult demographic, females exhibited markedly higher DALYs rates than males, with the largest disparity observed in the 80 to 84 age group (1515.01 for males vs 1856.67 for females per 1,00,000 population).

When comparing the 2021 data to the 1990 data, the DALYs rate for individuals aged 40 to 79 generally decreased, whereas a notable increase was observed in those aged ≥80. Among males, the population aged ≥75 years experienced higher DALYs rates in 2021 than in 1990. In females, the increase in DALYs numbers was more pronounced, particularly in those aged ≥85 years, rising from 3047.20 in 1990 to 3659.83 in 2021 (a 16.74% increase).

### 3.3. Global and China trends in LBMD burden (1990–2021)

**Figure 2.**
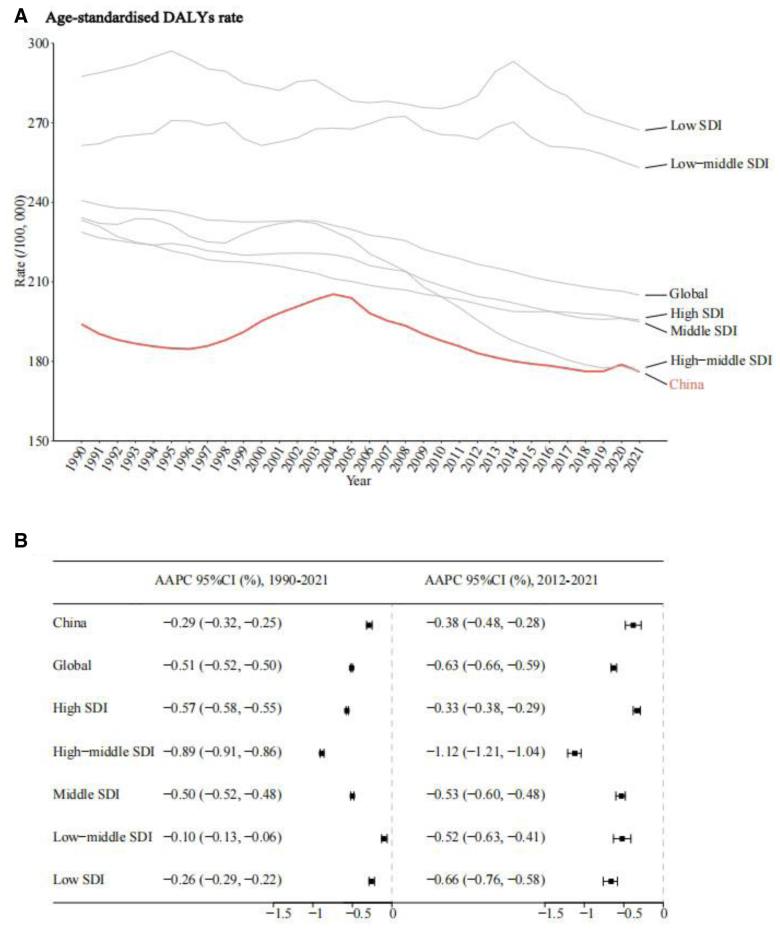
Summarizes the temporal trends and key statistical metrics of the LBMD burden from 1990 to 2021 across global, Chinese, and various socio-demographic index (SDI) regions. Panel (A) illustrates the trends in the age-standardized DALY rate for each region. Panel (B) provides a detailed statistical summary in table format, listing the average annual percentage change (AAPC) values alongside their 95% confidence intervals for 2 periods (1990–2021 and 2012–2021). The point estimates are denoted by black squares, with the accompanying horizontal lines representing the range of the confidence intervals. AAPC = average annual percentage change, DALY = disability-adjusted life year, LBMD = low bone mineral density, SDI = socio-demographic index.

Globally, the age-standardized DALYs rate for LBMD decreased from approximately 240 per 1,00,000 individuals in 1990 to approximately 210 per 1,00,000 in 2021, with an AAPC of −0.51% (95% CI: −0.52%, −0.50%; Fig. 2). In China, the rate declined from approximately 195 per 1,00,000 in 1990 to approximately 175 per 1,00,000 in 2021, with an AAPC of −0.29% (95% CI: −0.32%, −0.25%). However, this decline was accompanied by a temporary increase in the age-standardized DALYs rate between 1997 and 2005 and a brief rise in 2020.

From the perspective of different SDI regions, the age-standardized DALYs rate in the low SDI region remained the highest from 1990 to 2021, consistently exceeding 250 per 1,00,000 population, followed by the low-middle SDI region. In contrast, the high, middle, and high-middle SDI regions exhibited lower rates than the global averages. Notably, after 2016, the rates in the high and middle SDI regions converged, whereas the high-middle SDI region demonstrated a notably lower rate than the high and middle SDI regions since 2010. The AAPC for the high-middle SDI region from 2012 to 2021 was −1.12% (95% CI: −1.21%, −1.04%), indicating the fastest decline among all regions.

### 3.4. Future prediction trend of age-standardized DALY in China

**Figure 3.**
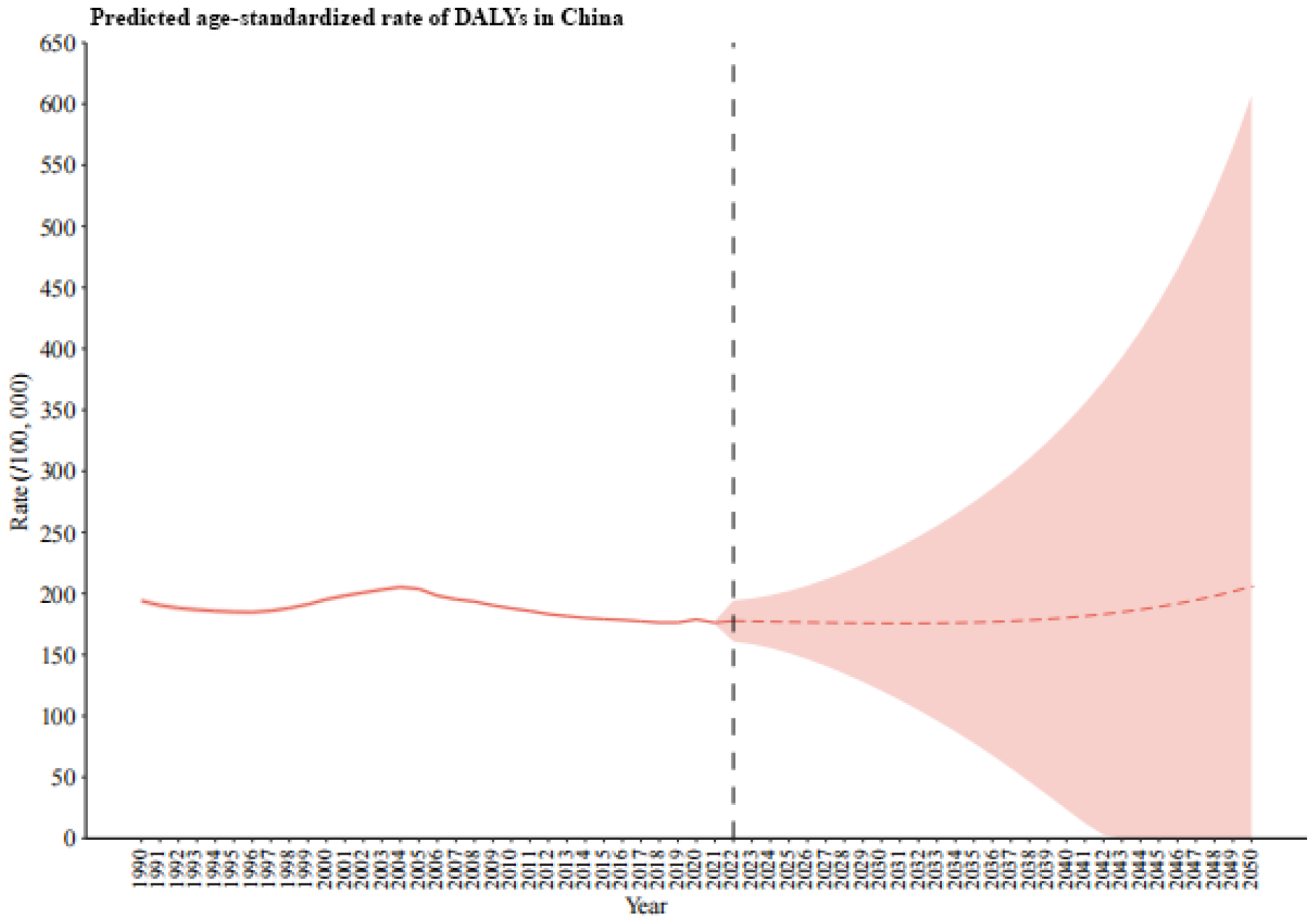
Displays the projected age-standardized DALY rates for LBMD in China, derived from a Bayesian age-period-cohort model. The forecast suggests a phase of relative stability until around 2040, after which a gradual upward trend is anticipated through to 2050. The accompanying shaded area clearly indicates the uncertainty intervals associated with the prediction.

Based on the BAPC projection model, the age-standardized DALY rate for LBMD in China is projected to remain relatively stable in the near term, until approximately 2040. Beyond this point, the model indicates a potential increasing trend, with a projected value of approximately 210 per 1,00,000 population by 2050 (Fig. 3).

## 4. Discussion

In 2021, the global burden of LBMD, measured in absolute DALYs, exceeded 17 million, whereas in China, it was approximately 3.5 million. Compared to 1990, China has experienced an overall decline in the DALYs rate; however, the burden remains markedly higher among the elderly population, particularly among those aged ≥80 years. Women in this age group are at a particularly high risk of developing LBMD-related disease. Globally, there is an overall downward trend in age-standardized DALYs, with the fastest decline observed in the high-middle SDI regions. In China, the age-standardized DALYs rate is projected to remain relatively stable in the near future, but a long-term rise is anticipated, necessitating heightened vigilance and proactive measures.

### 4.1. Elderly and women: high-burden groups for LBMD in China

This study indicates that in China, the older adult population, particularly postmenopausal women, bears a disproportionately high LBMD burden. The DALYs rate among the elderly, especially those aged ≥80 years, is notably elevated compared to younger cohorts. This disparity arises from age-related bone density decline,^[21]^ compounded by estrogen deficiency-driven pathophysiological cascades. Postmenopausal estrogendepletion accelerates osteoclast-mediated bone resorption and disrupts vitamin D metabolism by reducing renal 1α-hydroxylase activity, impairing the conversion of 25(OH)D to bioactive 1,25(OH)_2_D_3_, which is a critical regulator of intestinal calcium absorption and bone mineralization.^[22,23]^ These dual mechanisms synergistically exacerbate bone loss, rendering elderly women vulnerable to LBMD-related fractures, even at moderate vitamin D insufficiency levels.^[24]^

A multimodal intervention framework targeting high-risk LBMD populations should integrate synergistic hormonal, nutritional, and behavioral factors. Foundational measures include population-wide osteoporosis screening and calcium/vitamin D supplementation,^[25]^ with intensified protocols for high-risk subgroups.^[26,27]^ Menopausal hormone therapy (MHT) exerts dual osteoprotective mechanisms by compensating for estrogen deficiency (reducing osteoclast activity) and upregulating vitamin D receptor expression in osteoblasts.^[28]^ However, its application requires personalized risk stratification considering cardiovascular and oncological profiles.^[29]^ For individuals with contraindications to MHT, precision nutrition strategies utilizing phytoestrogens (e.g., soy isoflavones) can partially mimic estrogenic bone preservation, with genistein specifically enhancing vitamin D receptor-DNA binding affinity in osteocytes to amplify 1,25(OH)_2_D_3_-mediated transcriptional regulation under hypoestrogenic conditions.^[30,31]^ Exercise prescriptions should combine resistance training^[32]^ and yoga,^[33]^ supplemented by wearable device-enabled behavioral nudges to address adherence barriers.^[34]^ Public health initiatives must systematically advance health literacy programs addressing sleep chronobiology,^[35]^ oral microbiome-bone metabolism axis modulation,^[36]^ and ultraviolet exposure optimization, while integrating mental health support to counteract depression/anxiety-driven impairments in cutaneous 7-dehydrocholesterol photoconversion (the rate-limiting step for vitamin D3 synthesis) and treatment discontinuation risks via HPA axis hyperactivation.^[37]^

### 4.2. Declining LBMD burden with persistent regional inequalities

The study revealed that although the global burden of LBMD has shown an overall declining trend, the burden remains high in low and low-middle SDI regions. Socioeconomic status significantly influences the burden of LBMD,^[38]^ and urban-rural disparities persist.^[39]^ Regions with lower socioeconomic status generally exhibit lower awareness of osteoporosis,^[40]^ and the prevalence of osteoporosis among older women in rural areas is higher than that among urban populations.^[41]^ To reduce these inequalities and improve public health outcomes, key strategies include promoting regional economic development, increasing investment in healthcare resources, and optimizing the allocation of resources. For instance, addressing transportation barriers and implementing case management to improve healthcare access for rural patients,^[42]^ building a BMD reference database for a healthy adult population,^[43]^ and drawing on the strategic experiences of high SDI regions^[44]^ are all important. The increase in China’s age-standardized DALYs rate attributable to LBMD in 2020 may reflect the prolonged impact of COVID-19 on skeletal homeostasis,^[45]^ with pandemic-related mobility restrictions likely exacerbating risks through impaired ultraviolet B-dependent vitamin D synthesis and disrupted postfracture rehabilitation access. While these compound effects nominally explain the observed DALY trajectory perturbation, formal counterfactual mediation models are required to quantify pathway-specific contributions and distinguish direct viral pathogenesis from indirect behavioral sequelae of the disease.

### 4.3. Future trends and policy recommendations

Projections indicate that the burden of LBMD in China may increase after 2040, potentially linked to demographic changes in the population. Rising life expectancy has led to a growing proportion of the population aged ≥80 years, significantly intensifying pressure on healthcare and elderly care systems. Effectively utilizing limited medical resources to address the LBMD burden remains challenging. Studies suggest that the insidious nature of osteoporosis leads to its underestimation, and despite its high prevalence, it is often deprioritized in healthcare management.^[46]^ This highlights the need to strengthen early screening and preventive measures and to reallocate medical resources to meet changing demands.

In the future, innovative technological approaches could be integrated, such as using deep learning models to predict bone mineral density and support clinical decision-making,^[47]^ leveraging virtual reality technology to assist in exercise programs aimed at increasing bone density,^[48]^ and providing personalized nutritional guidance for high-risk populations within the context of metagenomics and precision medicine.^[49]^

## 5. Limitations

This study had several limitations that should be considered. First, the dependence on GBD secondary data introduces dual biases: symptomatic ascertainment thresholds likely underestimate the subclinical LBMD burden in populations without systematic screening programs, whereas incomplete coverage in low-resource settings compounds geographic selection biases. Second, the age-standardized DALY metric, despite its utility for cross-regional comparisons, obscures geriatric-specific burden gradients, particularly among octogenarians, in whom competing mortality risks modulate fracture outcomes. Third, the BAPC predictive framework’s assumption of stable secular trends inadequately captures 3 critical dynamics: nonlinear technological disruptions (e.g., anabolic osteoporosis therapies), abrupt demographic shifts (e.g., China’s 2023 fertility policy amendments), and pandemic-related behavioral perturbations affecting long-term bone health trajectories.

## 6. Conclusion

LBMD and related diseases impose a significant burden on both the Chinese and global populations. Women, the elderly, and populations in low-middle and middle SDI regions are particularly high-risk groups, warranting prioritization in future studies. To mitigate the burden of LBMD, future efforts should focus on strengthening prevention, early diagnosis, and comprehensive management strategies for LBMD. This includes promoting public health interventions, optimizing healthcare resource allocation, and leveraging innovative technologies to improve outcomes in at-risk populations.

## Author contributions

**Conceptualization:** Leiming Ge, Yao Liu, Xiaopan Li, Zheng Ye.

**Data curation:** Zhijuan Qi, Ling Shi, Xiaodong Wang.

**Formal analysis:** Leiming Ge, Zhijuan Qi.

**Investigation:** Leiming Ge.

**Methodology:** Yao Liu, Ling Shi, Jianling Song, Xiaopan Li.

**Project administration:** Zheng Ye.

**Software:** Jianling Song.

**Supervision:** Xiaopan Li, Zheng Ye.

**Validation:** Jianling Song, Xiaopan Li.

**Visualization:** Shuping Zheng.

**Writing – original draft:** Leiming Ge, Zhijuan Qi.

**Writing – review & editing:** Yao Liu, Xiaopan Li, Wenchang Jia, Zheng Ye.
